# Premature closure of the caudal calvarial midline suture is associated with scaphocephaly and ventriculomegaly in Boxer dogs

**DOI:** 10.3389/fvets.2025.1680045

**Published:** 2026-01-13

**Authors:** Daniela Farke, Blanche Guillier, Kathrin Büttner, Martin J. Schmidt

**Affiliations:** 1 Department of Veterinary Clinical Sciences, Small Animal Clinic, Justus-Liebig-University, Giessen, Germany; 2 Institute of Veterinary-Anatomy -Histology, and -Embryology, Justus-Liebig-University, Giessen, Germany; 3 Department of Biomathematics, Justus-Liebig-University, Giessen, Germany

**Keywords:** brachycephaly, canine, craniosynostosis, osteogenesis, skull

## Abstract

**Introduction:**

Boxer dogs exhibit a distinctive skull morphology resembling scaphocephaly in humans. This study investigates the status of skull sutures in Boxer dogs in comparison with other brachycephalic and mesocephalic breeds.

**Methods:**

Archival magnetic resonance images of the heads of 312 dogs with variable skull morphologies were examined. Sutures and synchondroses of the skulls were assessed as open or closed, and the presence or absence of ventricular dilation was noted.

**Results:**

A total of 160 dogs belonged to the mesocephalic group, 103 were brachycephalic, and 49 were Boxer dogs. Age was a predictor for closed sutures and synchondroses (*p* < 0.05). Sutures and synchondroses were more likely closed in brachycephalic compared to mesocephalic dogs (*p* < 0.0001). In addition, brachy- and mesocephalic dogs were less likely to show a closed sagittal suture (S4), parietointerparietal suture (S9) (*p* < 0.0001), and lambdoid sutures (*p* < 0.05) than Boxer dogs. Cranial index was higher in brachycephalic dogs compared to mesocephalic dogs and Boxer dogs and significantly differed among all groups (*p* < 0.05). Width/height index of the skull was significantly different among all groups and lowest in Boxer group (*p* < 0.05). Boxer dogs more likely experienced ventriculomegaly than the other breeds (*p* < 0.0001).

**Discussion/conclusion:**

The parietointerparietal and sagittal suture are more likely fused in Boxer dogs. A premature suture closure is most likely responsible for the Boxer dog’s unique skull morphology or scaphocephaly and an associated ventriculomegaly, which resembles a non-syndromical craniosynostosis in humans.

## 1 Introduction

**Figure 1:**
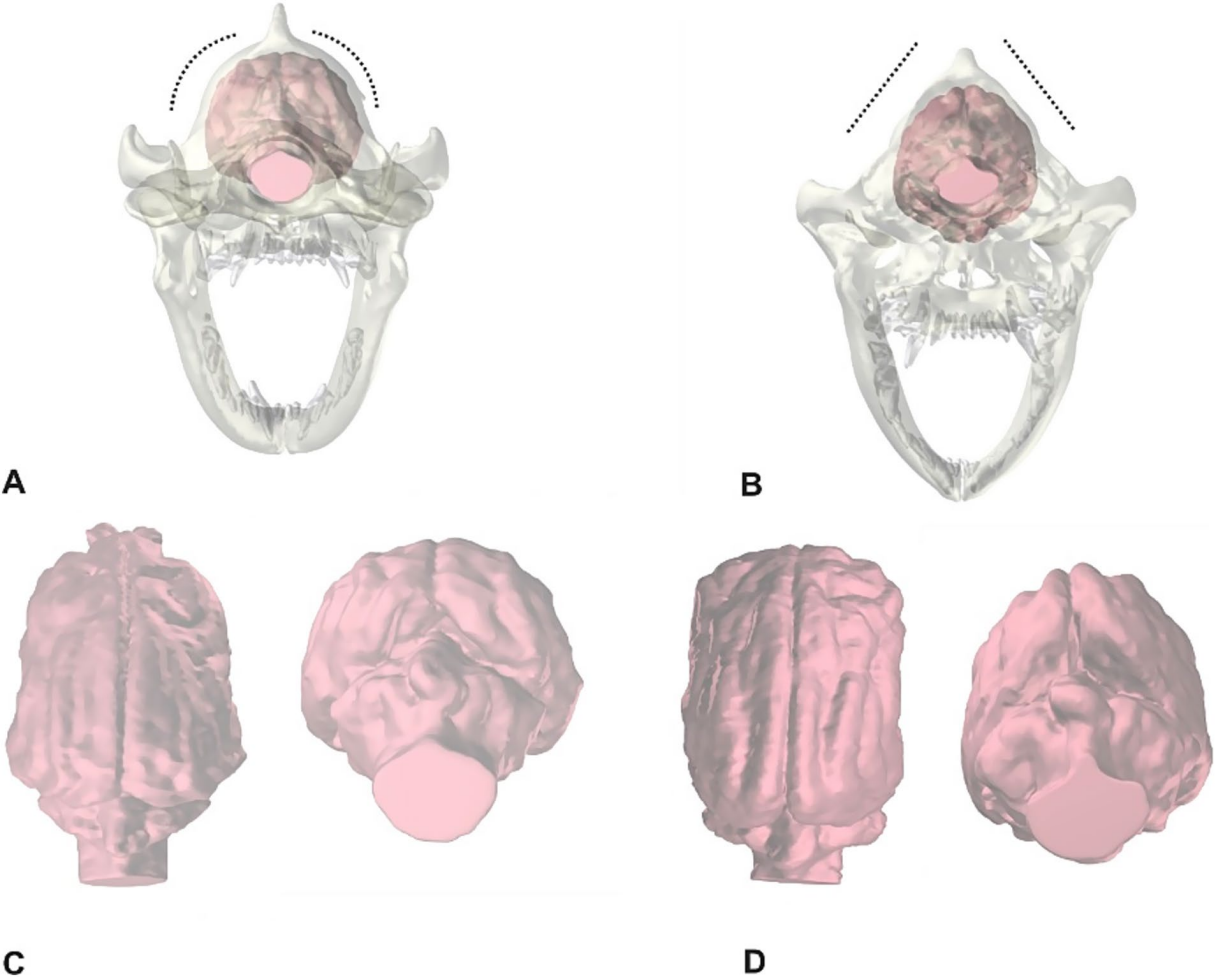
These pictures show a reconstruction of the skull **(A,B)** and brain parenchyma (pink) in dorsal and caudal transverse views **(C,D)** of a 3-year-old Golden Retriever **(A,C)** and a 2-years old Boxer dog **(B,D)**. The dotted lines in A and B highlight the differences of both skull shapes.

**Figure 2:**
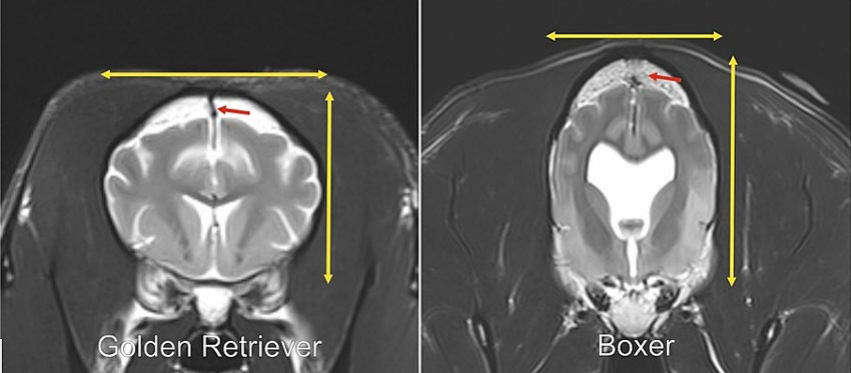
In the left and right picture is a T2-weighted transverse magnetic resonance image of the head at the level of the lateral ventricles and caudate nucleus of a 3 years old Golden Retriever (left) and a 2-years old Boxer dog (right). The red arrows point to the sagittal suture, which is clearly visible on the left and absent on the right picture. The yellow arrows point out the differences between the skull height and width of the Golden Retriever and the Boxer dog, with the Boxer dog showing an increased height and reduced width of the skull. A moderate enlargement of the lateral ventricles can be observed in the Boxer dog compared to the Golden Retriever.

The Boxer dog is a mastiff-type dog breed developed in Germany in the late 19th century (1). Since the times of its original development, the skull morphology has become increasingly modified towards a reduced length of the facial skeleton in relation to the neurocranium (2–6). The shortening of the skull length relative to its width causes a dome-shaped skull and a dorsal rotation and shortening of the facial bones that describe the brachycephalic phenotype (5, 6). Mesocephalic dogs show a greater longitudinal axis of the skull and a more narrowed skull width compared to brachycephalic breeds with prominent and long facial features (7). The skull conformation of the Boxer dog is traditionally classified as brachychephalic (8, 9). In contrast to this, a recent investigation described the Boxer dog correctly as airorhynch, which describes dorsal rotation/upward tilting of the palate relative to the cranial base (10). In true brachycephalic dogs, the longitudinal dimension of the neurocranium is reduced, whereas in modern Boxer dog the neurocranium has become progressively elongated compared to dogs of the same breed from the previous century (3). It was also noted that the skull vault in Boxer dogs appears tapered towards the dorsal aspect of the calvaria (Figures 1, 2). The usually convex hemispheres are more linear and almost arrow-shaped in Boxer dogs (Figures 1, 2) and show variable degrees of ventriculomegaly, which was suggested to be a normal anatomical variant (11).

Increasing evidence suggests that brachycephalic skull shapes and ventricular system dilation are more likely indicative of a pathological condition than a normal physiological variant (12–14). A premature closure of one or more skull sutures, which is referred to as craniosynostosis, was found to be associated with brachycephaly in dogs and cats (15–17). In both species, early closure of a skull suture is followed by secondary skull deformities that follow a predictable pattern. If the normal bone growth is inhibited in the orthogonal direction relative to the closed suture, a compensatory bone growth develops in parallel direction of the closed suture (18).

Given that the modern Boxer dog presents a longer but narrowed neurocranium, we hypothesized that a premature closure in the metopic (interfrontal) or sagittal suture occurs in Boxer dogs. We therefore examined the status of the neurocranial sutures in magnetic resonance images in a series of Boxer dogs, as well as brachycephalic and mesocephalic dogs.

## 2 Materials and methods

### 2.1 Animals

The archive of magnetic resonance imaging (MRI) scans of the Clinic for Small Animals at Justus Liebig University in Germany was searched for cranial studies of dogs aged 3 weeks to 15 years from 2017 to 2022. Age and sex were recorded. Animals with structural lesions affecting the skull and brain were excluded from the study. Animals were examined as part of a diagnostic procedure, and images were used retrospectively, which is not subject to institutional or governmental regulations in Germany.

A standard anesthetic protocol was used for the MRI examination and surgical procedure in each animal. Diazepam (0.5 mg/kg) was administered intravenously into a venous catheter (18, 20, or 22 gauge) placed in the right or left cephalic vein. Anesthesia was induced with propofol (2–4 mg/kg, IV). Dogs were endotracheally intubated, and anesthesia was maintained with 2% isoflurane in oxygen.

### 2.2 Magnetic resonance imaging

Imaging was performed with a 3.0 Tesla high field MRI scanner (Phillips Intera Gyroscan, Philips Healthcare, Hamburg Germany) or 1.5 Tesla high field MRI scanner (Siemens Verio, Siemens Healthcare, Erlangen Germany). The dogs were positioned in sternal recumbency. Images included at least sagittal and transverse T2-weighted images with a 2-mm slice thickness and a 0.2-mm slice interval (T2-Turbospin echo, echo time 120 ms, repetition time 2,900 ms). T1-FFE weighted dorsal and transversal images with a slice thickness of 1 mm were obtained. Field of view was 120 mm× 120 mm in small dogs and 210 mm× 210 mm in large dogs. Matrix was 288 × 288 in small dogs and 384 × 384 in large dogs, leading to a pixel size between 0.625 mm× 0.625 mm and 0.54 mm× 0.54 mm.

### 2.3 Image analysis

**Figure 3:**
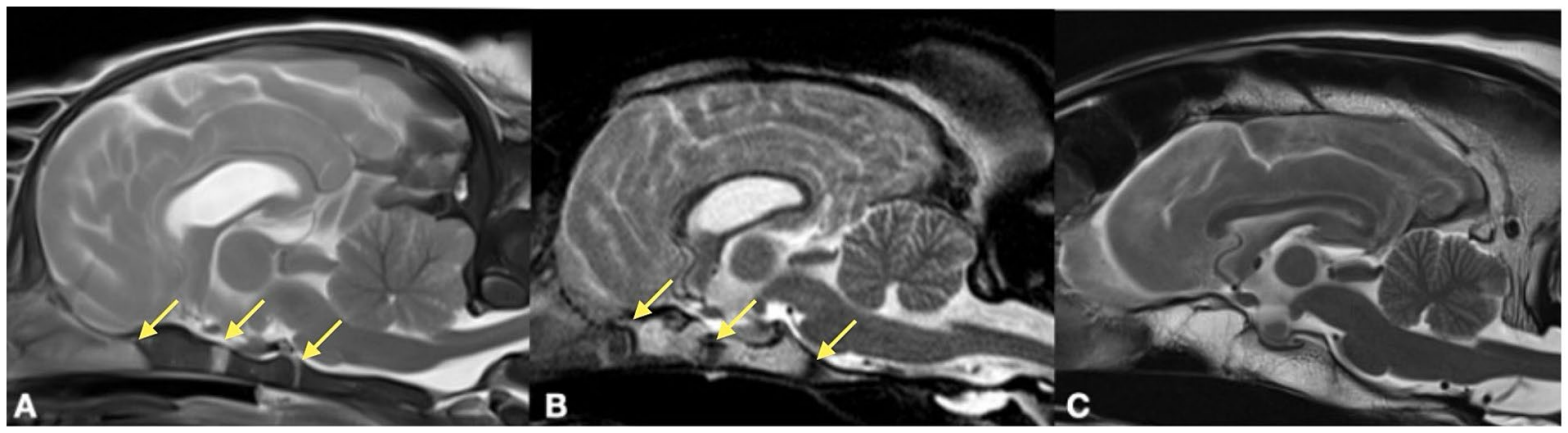
T2-weighted midsagittal MRI of Boxer dogs at the level of the skull base at the age of 1 month **(A)**, 12 months **(B)** and 108 months **(C)**. The skullbase snychondroses (yellow arrows) are visible as well-defined hyperintense signals with hypointense borders **(A)** and as a hypointense signal zone in a more mature dog **(B)**. In contrast there is a loss of signal and replacement by bone in the old Boxer dog **(C)** where a clear synchondrosal structure is not detectable.

**Figure 4:**
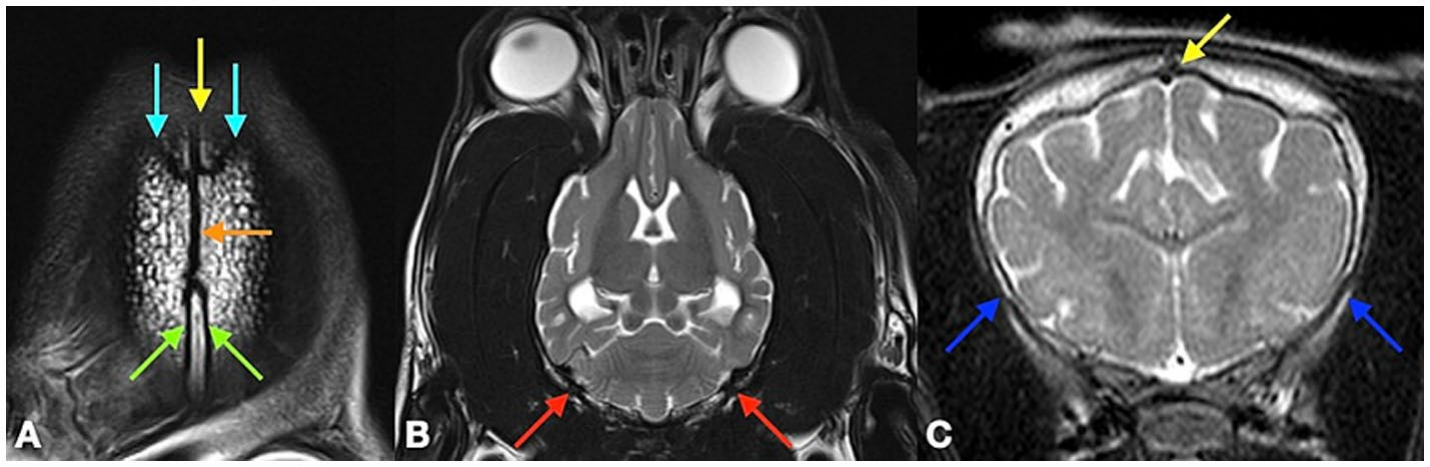
T2 weighted MRI pictures of an 8-month-old dog in a dorsal plane at the level of the calvarial bones **(A)**, in a dorsal plane at the level of the midbrain tectum **(B)** and a transversal plane at the level of the caudate nuclei **(C)**. Picture **A** shows the metopic suture (S1 = yellow arrow), the left and right coronal sutures (S2, S3 = bright blue arrows), the sagittal suture (S4 = orange arrow) and the parietointerparietal sutures (S9 = green arrows). Picture **B** point out the left and right lambdoid sutures (S10, S11 = red arrows) and picture **C** shows the sagittal sutures (S1 = yellow arrow) and the left and right sphenofrontal sutures (S5, S6 = blue arrows).

All images from 312 dogs were retrieved from the relevant picture archiving system and evaluated by a board-certified neurologist (DF). The analysis was performed with anonymized and randomized image data sets. The observer was blinded to age and breed of the dogs. The open or closed status of the following cranial desmal growth plates were examined along their visible length by using the different MRI planes: interfrontal (metopic) suture (S1); fronto-parietal (coronal) sutures (S2/3); sagittal suture (S4); spheno-frontal suture (S5/6); sphenoparietal suture (squamosal) suture (S7/8); parieto-interparietal suture (S9); and lambdoid suture (S10/11). Synchondondroses included sphenoethmoidal (SE) synchondrosis, intersphenoidal (IS) synchrondrosis, and sphenooccipital (SO) synchondrosis (Figures 2–4). An open suture was defined as a hypointense signal interruption of the hyperintense calvarial bone marrow signal in T2-weighted images (Figures 2, 4). An open synchondrosis was identified by the presence of a broad hyperintense signal area (cartilage) with adjacent, well-defined hypointense margins (endplates) in very young specimens, or by a distinct hypointense signal interrupting the bone structure in adult dogs in T2-weighted images (Figure 3). A closed suture or synchondrosis was defined as a lack of a hypointense signal within the hyperintense bony structures in T2-weighted images (Figures 2, 3). Partial closure was defined as bony bridges within the suture or synchondrosis, visible as a partial hypointense signal of a suture within the hyperintense bone, or as a hyperintense bone signal within the hypointense cartilage signal of the synchondrosis, with a narrowing of the synchondrosal endplates.

Cranial length and width were measured in the dorsal plane at the level of the tectum of the midbrain. Cranial height was measured in the midsagittal plane from the skull base to the calvarium at the level of the hypophysis. The presence or absence of ventricular dilation (ventriculomegaly) was noted for each dog. The focus was based on the dilation of the lateral and third ventricles. A physiological lateral ventricle was considered to have a drop-like or triangular shape of the body of the lateral ventricle that is followed by the temporal horn as a slim line. Any further enlargement was considered ventriculomegaly, as suggested in a ventricular grading by Czerwik et al. (19). Dogs that showed MRI signs indicative of clinically relevant internal hydrocephalus or brain atrophy were excluded from the study. These MRI signs were periventricular edema, flattening or widening of gyri and sulci, disruption of the internal capsule, and deformation of the interthalamic adhesion (20, 21).

### 2.4 Statistical analysis

All statistical tests were selected and performed by a professional statistician (KB), using commercially available software (Graph Pad Prism 4.0, Graph Pad Software Inc., San Diego, California). Sutures were categorized as open or closed. Partially closed sutures were counted as closed sutures for statistical analysis. Only sutures that were clearly identifiable as open or closed were included in the statistical analysis. Dogs were grouped into mesocephalic controls, brachycephalic controls, and Boxer dogs. In the first step, a multiple logistic regression was performed to test the global effect of age and sex on closure of the synchondroses within each group. In a second step, a multiple logistic regression was performed to compare the three groups of dogs, considering the effect of age and sex. In the last step, mesocephalic, brachycephalic, and Boxer dog were compared pairwise by means of a Wald-test and a Chi-square statistic. An exact Pearson Chi-square test followed by pairwise analysis and Bonferroni correction was performed to assess the presence of ventriculomegaly among the three groups of dogs. An analysis of variance was performed with cranial index (width/length) and width/height index as the dependent variables and group as the fixed variable. The residuals were normally distributed. The pairwise comparisons were adjusted with the Bonferroni correction. A pairwise t-test was performed to evaluate effects of these cranial indices on the presence of ventriculomegaly. The ranges of all Odds ratios and medians are given in parentheses after the value. For all tests, a *p* value of ≤0.05 was considered significant.

## 3 Results

### 3.1 Animals

A total of 2,702 sutures and 935 synchondroses were examined; 730 sutures and one synchondrosis were excluded due to poor visualization. There were 126 female and 186 male dogs. Of the 312 dogs examined, 49 were Boxer dogs, 160 were mesocephalic, and 103 were brachycephalic breeds (Table 1). The median age of the Boxer group was 84 months (range 0.9–132 months), of the mesocephalic group 60 months (range 1.3–180 months), and of the brachycephalic group 48 months (range 3.8–144 months).

**Table 1:** The table shows the different breeds and their distribution among the mesocephalic and brachycephalic group.

Breed	Nr.
Mesocephalic (160)	
Labrador retriever	22
German shepherd	15
Australian shepherd	14
Golden retriever	13
Mixed breed	9
Border collie	9
Beagle	7
Bavarian mountain dog	6
German wire haired pointer	5
Bernese mountain dog	5
Husky	4
Rhodesian Ridgeback	4
Weimaraner	4
Appenzell mountain dog	4
Belgian shepherd	3
Dachshund	3
Magyar viszla	3
West highland white terrier	3
Akita inu	2
Bearded collie	2
Dobermann pinscher	2
Galgo espanol	2
Irish red setter	2
Jack russel terrier	2
Miniature schnauzer	2
Pudel	2
Whippet	2
Airdale Terrier	1
Cocker spaniel	1
Collie	1
Dalmatian	1
Eurasier	1
Flat coated retriever	1
Hovawart	1
Leonberger	1
Portuguese waterspaniel	1
Brachycephalic (103)	
French bulldog	38
English bulldog	22
Cavalier king charles spaniel	11
Chihuahua	9
Pug	7
Pomeranian	6
Boston terrier	3
Mixed breed	3
ShiTzu	3
Pekinese	1

### 3.2 Influence of age and sex on closure state of sutures and syndrondroses

Age was found to be a predictor of suture closure in most growth centers examined. The Odds ratio for the metopic suture (S1) was 4 (1.3–12.2) (*p* = 0.0138), for the coronal sutures (S2/3) 8.4 (3.3–21.4) (*p* < 0.0001) and 7.1 (2.9–17.7) (*p* < 0.0001), for sagittal suture (S4) 6.2 (2.6–14.8) (*p* < 0.0001), for sphenofrontal sutures (S5/6) 8.4 (range 1.5–47.1) (*p* = 0.016), for squamosal sutures (S7/8) 4.1 (1.1–15.1) (*p* = 0.0338) and 4.4 (1.2–16.5) (*p* = 0.0281), and for lambdoid sutures (S10/11) 4.1 (2.0–8.3) (*p* < 0.0001) and 4.5 (2.2–9.3) (*p* < 0.0001). The Odds ratios for skull base synchondroses were 6 (2.8–12.5) (*p* < 0.0001) for sphenooccipital (SO), 24 (9.7–62.3) (*p* < 0.0001) for intersphenoidal (IS), and 6 (2.9–12.1) (*p* < 0.0001) for sphenoethmoidal (SE) synchondrosis that are most likely closed at older age (Table 2). No significant influence of age on suture closure was found for parietointerparietal suture (S9) (*p* > 0.05) (Table 2). A difference in sex was identified for closure of intersphenoidal synchondrosis (IS) with an Odds ratio of 1.9 (95% confidence interval = 1.1–3.4) (*p* = 0.0277) and parietointerparietal sutures (S9) with an Odds ratio of 2.3 (95% confidence interval = 1.1–4.8) (*p* = 0.0229) for female dogs with closed synchondrosis or suture compared to males (Table 2).

**Table 2:** The table shows the Odds ratio (OR) for the finding of a closed suture or synchondrosis with age per year, compared between brachy- and mesocephalic dogs, brachycephalic dogs and Boxer dogs and mesocephalic dogs and Boxer dogs.

Sutures and synchondroses	Nr. of obs.	OR and CI 95% (age)	*p*-value	OR and CI 95% (brachy- vs. mesocephalic)	*p*-value	OR and CI 95% (brachycephalic vs. Boxer)	p-value	OR and CI 95% (mesocephalic vs. Boxer)	*p*-value
Metopic (S1)	311	4 (1.3–12.2)	**0.0138**	14.9 (4.945.3)	**<0.0001**	0.7 (0.3–1.5)	0.31	0.05 (0.01–0.1)	**<0.0001**
Coronal (S2)	309	8.4 (3.3–21.4)	**<0.0001**	20.4 (9.7–43)	**<0.0001**	0.8 (0.3–1.7)	0.5039	0.04 (0.02–0.1)	**<0.0001**
Coronal (S3)	309	7.1 (2.9–17.7)	**<0.0001**	17.7 (8.5–36.9)	**<0.0001**	0.7 (0.3–1.5)	0.3453	0.04 (0.02–0.1)	**<0.0001**
Sagittal (S4)	311	6.2 (2.6–14.8)	**<0.0001**	3.9 (2.1–7.5)	**<0.0001**	0.04 (0.01–1)	**<0.0001**	0.01 (0.002–0.03)	**<0.0001**
Sphenofrontal (S5)	113	8.4 (1.5–47.1)	**0.016**	14.7 (4.8–44.9)	**<0.0001**	5 (0.8–32.7)	0.0928	0.3 (0.1–2.1)	0.2505
Sphenofrontal (S6)	113	8.4 (1.5–47.1)	**0.016**	14.7 (4.8–44.9)	**<0.0001**	5 (0.8–32.7)	0.0928	0.3 (0.1–2.1)	0.2505
Squamosal (S7)	165	4.1 (1.1–15.1)	**0.0338**	10 (4.1–24.4)	**<0.0001**	9.5 (1.9–48.4)	**0.0067**	1 (0.2–4.7)	0.9454
Squamosal (S8)	164	4.4 (1.2–16.5)	**0.0281**	11.1 (4.5–27.4)	**<0.0001**	5.9 (1.4–24.4)	**0.0151**	0.5 (0.1–2.2)	0.3784
Parietointerparietal (S9)	303	2.2 (0.8–5.7)	0.1121	14.6 (4.8–44.6)	**<0.0001**	0.1 (0.07–0.3)	**<0.0001**	0.01 (0.003–0.03)	**<0.0001**
Lambdoid (S10)	302	4.1 (2–8.3)	**<0.0001**	7.7 (4.1–14.5)	**<0.0001**	0.3 (0.07–1)	**0.0462**	0.03 (0.001–0.1)	**<0.0001**
Lambdoid (S11)	302	4.5 (2.2–9.3)	**<0.0001**	7.9 (4.2–15)	**<0.0001**	0.2 (0.03–0.8)	**0.0222**	0.02 (0.004–0.1)	**<0.0001**
Spenooccipital (SO)	312	6 (2.8–12.5)	**<0.0001**	4.5 (2.5–8.2)	**<0.0001**	26.8 (10.4–69.1)	**<0.0001**	6 (2.6–13.9)	**<0.0001**
Intersphenoidal (IS)	311	24 (9.7–62.3)	**<0.0001**	16.9 (7.9–36)	**<0.0001**	2.1 (0.8–5.4)	0.1452	0.1 (0.05–0.3)	**<0.0001**
Sphenoethmoidal (SE)	312	6 (2.9–12.1)	**<0.0001**	4.6 (2.6–8.2)	**<0.0001**	0.4 (0.2–1)	**0.0447**	0.08 (0.03–0.2)	**<0.0001**

Confidence intervals of 95% (CI 95%) and p-values are provided for each OR. Significant values are highlighted in bold letters and differences of Boxer dogs compared to both meso- and brachycephalic groups are highlighted in red. Sutures are listed as following: S1 = metopic suture, S2 = left coronal suture, S3 = right coronal suture, S4 sagittal suture, S5 = left sphenofrontal suture, S6 = right sphenofrontal suture, S7 = right squamosal suture, S8 = left squamosal suture, S9 = parietointerparietal suture, S10 = left lambdoid suture, S11 = right lamdboid suture, SE = sphenoethmoidal synchondrosis, IS = intersphenoidal synchondrosis and SO = sphenooccipital synchondrosis.

### 3.3 Influence of skull conformation on closure state of sutures and syndrondroses

All sutures and synchondroses were more likely to be closed in brachycephalic dogs than in mesocephalic dogs (*p* < 0.0001) (Table 2). In contrast, there was a significant difference between brachycephalic dogs and Boxer dogs and between mesocephalic dogs and Boxer dogs. Boxer dogs were more likely to show closed metopic suture (S1), coronal sutures (S2/3), sagittal suture (S4), parietointerparietal suture (S9), lambdoid sutures (S10/11), and overall skull base synchondroses (SE, IS, SO) compared to mesocephalic dogs. Brachy- and mesocephalic dogs were less likely (*p* < 0.0001) to show a closed sagittal suture (S4), with Odds ratios of 0.04 (0.01–0.10) and 0.01 (0.002–0.030), respectively, than Boxer dogs (Table 2; Figure 2). In addition, brachy- and mesocephalic dogs were also less likely (p < 0.0001) to show a closed parietointerparietal suture (S9), with Odds ratios of 0.1 (0.07–0.30) and 0.01 (0.003–0.030), respectively, than Boxer dogs (Table 2).

Coronal sutures (S2/S3), with an Odds ratio of 0.04 (0.02–0.10), were less likely (*p* < 0.0001) closed in mesocephalic dogs than in Boxer dogs, whereas no difference was detected between the brachycephalic and Boxer groups (Table 2). Squamosal sutures (S7/8), with Odds ratios of 9.5 (1.9–48.4) (*p* = 0.0067) and 5.9 (1.4–24.4) (*p* = 0.0151), were more likely closed in brachycephalic dogs than in Boxer dogs. Lambdoid sutures (S10/11), with Odds ratios of 0.3 (0.07–1.00) (*p* = 0.0462) and 0.2 (0.03–0.80) (*p* = 0.0222), respectively, were less likely closed in brachycephalic dogs than in Boxer dogs (Table 2).

### 3.4 Influence of skull morphology on the presence of ventriculomegaly

**Figure 5:**
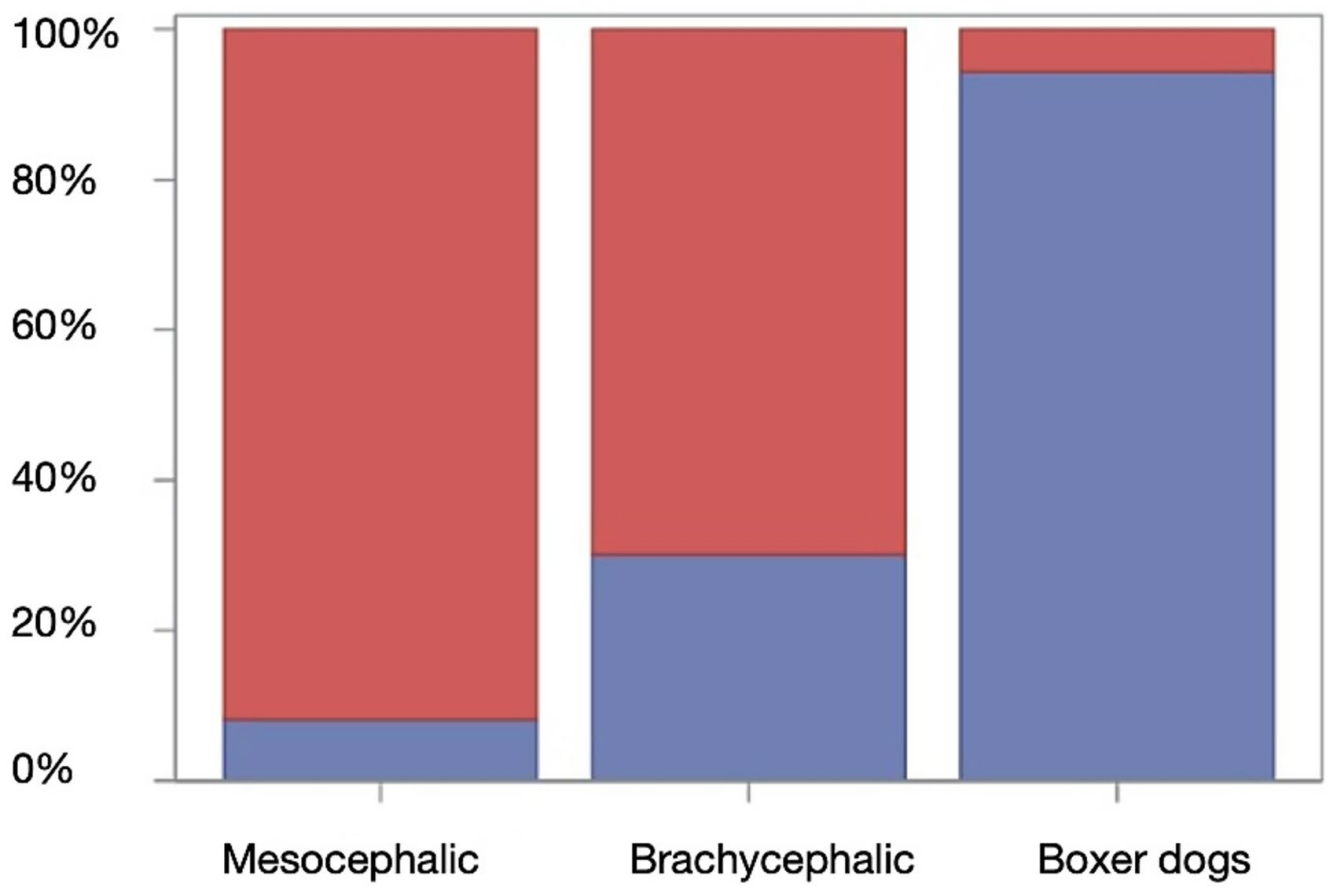
Box Plot showing the percentage of frequency for the presence (blue) or absence (red) of ventriculomegaly among the different groups.

Ventriculomegaly was present in nine mesocephalic dogs, in 72 brachycephalic dogs, and in 45 Boxer dogs. Specifically, mesocephalic dogs (9/160) showed significantly less (*p* < 0.0001) ventricular dilation compared to brachycephalic dogs (72/103) and Boxer dogs (45/49). Ventriculomegaly was more frequently observed (*p* = 0.0102) in Boxer dogs than in brachycephalic dogs (Figure 5).

**Figure 6:**
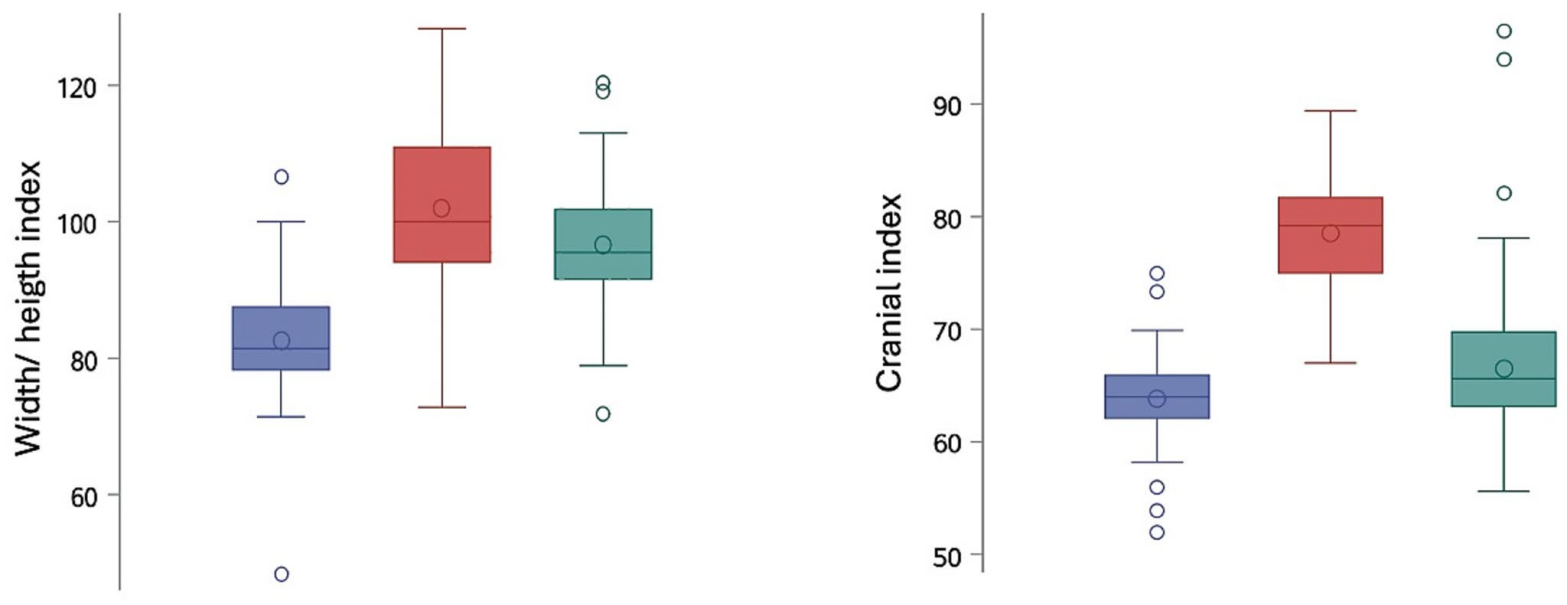
Box and whisker plots showing the width/ height (left) and cranial (right) index measurements among Boxer dogs (blue), brachycephalic (red) and mesocephalic group (green).

The median cranial index was 65.6 (55.6–96.5) for the mesocephalic group, 79.2 (67.0–89.4) for the brachycephalic group, and 64 (52–75) for the Boxer dogs (Figure 6). There was a significant difference in all pairwise comparisons (mesocephalic vs. brachycephalic (*p* < 0.0001), brachycephalic vs. Boxer dog (*p* < 0.0001), mesocephalic vs. Boxer dog (*p* = 0.0065)). Dogs with ventriculomegaly had a significantly higher (*p* < 0.0001) cranial index (median 74.7, 52.0–89.4) than dogs with normal ventricular dimensions (median 67, 55.6–96.5). Significant differences in median width/height index were detected among all groups (*p* < 0.0001). The median width/height index of the mesocephalic group was 95.5 (71.8–120.5), whereas it was 100 (72.8–128.3) for the brachycephalic group and 81.4 (48.3–106.6) for the Boxer group (Figure 6). The presence of ventriculomegaly was not significantly associated with the width/height index (*p* = 0.2529).

## 4 Discussion

Synchondroses and sutures are cranial growth centers that allow expansion of the brain and the cranial cavity. An increasing body of evidence suggests that general head morphology in dogs may be substantially influenced by the function of these growth plates (15, 16). A premature fusion of one or more cranial sutures may result in characteristic shortening of the cranial cavity and facial bones in brachycephalic dogs (15, 16, 22–24). Ethical concerns about breeding brachycephalic animals would arise if a pathological growth disorder were the basis of their head morphology. The ongoing study of cranial sutures and synchondroses in dogs therefore merits more serious consideration. Non-invasive diagnostic imaging methods, such as MRI and computed tomography (CT), are the primary imaging techniques used to evaluate the condition of the cranial sutures and synchondroses in children (25, 26). They have also been successfully used to investigate cranial growth centers in dogs (15). The use of MRI or CT permits the investigation of a large cohort of dogs and would also allow monitoring the temporal behavior of a single suture in the same animal. The use of CT offers a higher sensitivity regarding the evaluation of the calvarial sutures compared to MRI studies. There are differences among the visibility of the sutures depending on their location, structure, and bone thickness. In our study, 730 sutures were excluded from further analysis due to poor visualization in MRI. The dorsal calvarial sutures, such as the S1, S2/3, and S4, show a high sensitivity that is similar in MRI (73.9–95.8%) and CT (76.9–88.5%) and were most often visible in this study. On the other hand, S5/6, S7/8, and S10/11 offer a lower sensitivity of 73.7–87.5% in CT and even lower sensitivity of 50.0–73.1% in MRI and were less often visualized in this study, resulting in higher numbers of exclusions for these sutures. These findings are also dependent on the experience of the observer (27). In our study, all sutures and synchondroses were evaluated by an experienced board-certified neurologist, but the use of MRI is a limitation regarding the evaluation of S5/6, S7/8, and S10/11. Synchondroses, on the other hand, can be observed in MRI and CT with similar high sensitivities, which explains why only one synchondrosis was excluded due to poor visualization in this study. Another limitation is the use of two different MRI scanners with a magnetic strength of 1.5 and 3.0 Tesla, which might also have an impact on the visualization of the sutures. Higher sensitivity might be achieved by using a stronger magnetic field or a CT to improve visualization of more sutures.

For the assessment of synchondrosal status, both CT and MRI show good sensitivity and specificity (27). The large size of synchondroses, as well as the consistent change of signal from cartilage to bone tissue, accounts for this result. However, not all sutures can be assessed equally well (27). SE synchondrosis has a lower sensitivity compared to SO and IS synchondroses in both CT and MRI (27). The microanatomy of this synchondrosis differs from the others and is more difficult to define within the cranial base as it is not always straight but rather crescent-shaped (26). The transition to the air-filled nasal cavity blurs the contrast between the synchondrosis and the medullary cavity that allows good visualization of the other synchondroses.

There was a significant association between the closed status of the sutures and the skull base synchondroses with increasing age among all groups. However, the parietointerparietal suture did not show any dependency on age in suture closure, which might indicate that some calvarial sutures stay open for an entire lifespan as in humans (28). Another interesting finding was that the IS synchondrosis and the parietointerparietal suture were more likely to be closed in female dogs than in males. This is also in line with human studies, where sex differences were identified for the skull base synchondroses, which close at age 11–14 years in women and at age 13–16 years in men (29, 30). Differences in the onset of puberty are suggested as the cause of the difference in humans (30). However, differences in sex hormones such as estrogen and their effect on osteoblast function must be also assumed to cause the difference in dogs. Estrogen receptors are expressed in osteoblasts, osteoclasts, and osteocytes and therefore play an essential role in bone regulation and interfere with multiple signaling ways that increase oesteogenesis (31–33). Due to the retrospective character of our study, neuter status or age at neutering were not assessed, which is a limitation of this study. Early neutering influences growth plate closure and should be evaluated in a prospective study, together with the determination of suture closure (34–36).

Differences in suture closure were observed between meso- and brachycephalic dogs, with brachycephalic dogs more likely to show overall closed suture state. Furthermore, IS and SE synchondroses were also more often closed in brachycephalic than mesocephalic dogs. These findings explain the cranial conformations of brachycephalic dogs with an increased width and reduced length of the cranial vault and skull base. These differences were most evident for the skull base synchondrosis, which is in line with other studies that show significant differences between brachy- and mesocephalic dogs for the SO synchondrosis, which was already proven to close within 9 months in brachycephalic breeds compared to 13 months in mesocephalic breeds (15). There were only weak significant differences for sphenofrontal, squamosal, and lamboid sutures in the comparisons among the groups. This might be due to a relatively small sample size and more missing data values among these data as these sutures are often harder to evaluate than those on the top of the cranial vault (27). CT has higher sensitivity compared to MRI, especially for these sutures (27). Additional CT examination could therefore help to assess sutures that are less visible in MRI and reduce missing data due to poor visualization.

Compared to meso- and brachycephalic dogs, Boxer dogs were more likely to show closure of the midline sagittal and parietointerparietal sutures. The metopic suture was also more frequently closed in Boxer dogs than in mesocephalic dogs but not compared to brachycephalic dogs. Since only premature closure influences skull morphology, the higher median age of the dogs in our study limits conclusions about whether metopic suture closure occurs earlier in Boxer dogs.

Due to the inhibition of the longitudinal skull growth centers, a compensatory bone growth originating from the growth centers of the bilateral coronal suture develops in parallel direction of these closed sutures, resulting in a scaphocephalic skull morphology (18). This is in alignment with our hypothesis that the modern Boxer dog presents a longer but narrowed neurocranium due to a premature closure in the sagittal and caudally aligning parietoparietal suture. Furthermore, coronal sutures and lambdoid sutures are more likely closed in Boxer dogs than in mesocephalic dogs. An explanation might be the higher median age of the boxer group (7 years) and a given higher probability of sutures being closed at this time. Another explanation is that these findings also account for the Boxer dog being a brachycephalic skull type with pronounced calvarial width as well. Despite the relatively high ages of all groups, the sagittal suture in Boxer dogs is closed compared to meso- and brachycephalic dogs in which this suture remains in an open state for a longer period.

There were significant differences in the cranial index and the width/height index among all groups. These differences reflect the impact of premature suture closure on skull morphology and resulting phenotypic characteristics among the breeds (15–18, 22, 26). Our study points out that ventricular dilation is rarely observed in mesocephalic breeds but is a common finding among brachycephalic phenotypes and even more common in Boxer dogs. This is in line with other studies comparing different grades of brachycephaly in Persian cats and their association with ventricular dilation (17). It is interesting to note that the median cranial index of mesocephalic dogs and Boxer dogs is similar and that the cranial index of brachycephalic skull conformations is higher than in the other groups. This could indicate that the time of coronal suture closure in Boxer dogs occurs after completion of the cranial vault growth and shaping and does not contribute to an increased skull width and dome shape as in brachycephalic breeds. The time point of closure of the coronal suture could not be assessed in our study due to a relatively high median age among the groups. However, the coronal suture has been found to prematurely close at age 0.3 months in brachycephalic cats compared to mesocephalic cats (17). Therefore, a determination of the time point of suture closure would be helpful to further evaluate these differences in dogs.

The cranial width/height index shows a similarity between meso- and brachycephalic dogs, which have a larger index compared to Boxer dogs. This finding highlights the impact of the sagittal suture on the width growth of the cranial vault and indicates early closure of this suture with an impact on skull growth in Boxer dogs. The premature closure of the sagittal suture results in compensatory growth originating from the surrounding coronal sutures and probably squamosal and sphenofrontal sutures, resulting in elongation and increasing height of the cranial vault as described in humans (18). A higher cranial index was associated with the presence of ventriculomegaly. The cranial width/height index was not associated with the presence of ventriculomegaly, but Boxer dogs were more prone to this finding than the other two groups. These differences between the association of ventriculomegaly and the cranial index and the width/height index might be due to the different sample sizes of the groups. If there was a larger sample size in the Boxer group, a significant association of ventriculomegaly and the width/height index might have been detected, but this must be further investigated. Volumetric studies or a grading of the ventricular dilation were not performed in our study, which solely investigated the presence or absence of ventricular dilation of the lateral third or fourth ventricle. Further studies including volumetric measurements and a detailed analysis of which ventricles are affected might provide further insights about the anatomical changes in brachycephalic breeds and Boxer dogs specifically.

Scaphocephaly is defined as the premature closure of the sagittal suture and is the most frequent non-syndromic craniosynostosis in humans (37). Compensatory skull growth produces uniform longitudinal elongation with frontal and occipital bossing and secondary head deformation. The general aspect of the skull is “boat-shaped” with a narrow skull and cranial base and relatively normal facial development (37). In humans, the end point of cranial vault growth is determined upon fusion of the sutures in the third decade of life, but there can be considerable variability in closure rates (28, 38). Cranial sutures in mammals do not necessarily fuse when growth stops or slows down, suggesting that they have an additional role (39). The transformation of the sutural structure allows flexibility and energy absorption in the skull bones and reduces the risk of skull fractures in nature, which is why they remain in mature animals (40, 41). The fact that the end of sutural development in dogs is not necessarily determined by fusion demonstrates the impossibility to determine the physiological end of bone growth in the suture based on imaging techniques. Pathological craniosynostosis, on the other hand, might be diagnosed using CT or MRI (17, 21).

Our main limitation was the lack of histological sections for definitive proof of suture closure. CT scans would have been helpful to identify some sutures that are harder to visualize in MRI and would have therefore increased the number of samples for each suture (27). Another limitation might have been the high median age of our groups, which makes the identification of premature suture closure difficult. On the other hand, the broad age variation shows that some sutures, such as the metopic and sagittal sutures, stay open for a long time in brachy- and mesocephalic breeds. However, a larger sample size in each group would have helped to determine a closure time for each suture and skull type as well as it might have produced more reliable results regarding the association of the indices and the presence of ventriculomegaly.

## 5 Conclusion

The parietointerparietal and sagittal sutures are more likely fused in Boxer dogs compared to brachy- and mesocephalic dogs. A premature suture closure is most likely responsible for the specific long and narrow skull morphology, or scaphocephaly, and associated ventriculomegaly in Boxer dogs, which resembles non-syndromical craniosynostosis in humans.

## Data Availability

The raw data supporting the conclusions of this article will be made available by the authors, without undue reservation.
